# Corrigendum to “Novel Hybrid Gels Made of High and Low Molecular Weight Hyaluronic Acid Induce Proliferation and Reduce Inflammation in an Osteoarthritis *In Vitro* Model Based on Human Synoviocytes and Chondrocytes”

**DOI:** 10.1155/2020/7530149

**Published:** 2020-07-25

**Authors:** Antonietta Stellavato, Valentina Vassallo, Annalisa La Gatta, Anna Virginia Adriana Pirozzi, Mario De Rosa, Giovanni Balato, Alessio D'Addona, Virginia Tirino, Carlo Ruosi, Chiara Schiraldi

**Affiliations:** ^1^Department of Experimental Medicine, Section of Biotechnology, Medical Histology and Molecular Biology, University of Campania “Luigi Vanvitelli”, Naples, Italy; ^2^School of Medicine and Surgery “Federico II” of Naples, Department of Public Health, A.O.U. Federico II of Naples, Via S. Pansini, 80131Naples, Italy

In the article titled “Novel Hybrid Gels Made of High and Low Molecular Weight Hyaluronic Acid Induce Proliferation and Reduce Inflammation in an Osteoarthritis In Vitro Model Based on Human Synoviocytes and Chondrocytes” [1], there was an error in Figure 6. In the part of Figure 6(a), “HCC – 24h” is incorrect. The corrected figure is shown below, and is listed as Figure 1:

## Figures and Tables

**Figure 1:**
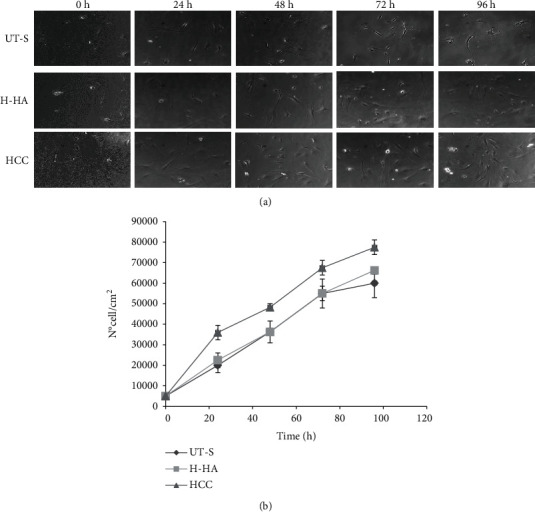
Human synoviocytes proliferation assay. (a) Pictures of cells at experimental times: 24, 48, 72, and 96 h; the proliferative properties of HCC were compared to the single H-HA and untreated synoviocytes (UT-S). (b) Cell growth curves were obtained through the Image-Pro Plus 1.5 software (Media Cybernetics).
